# Mycobacterium Time-Series Genome Analysis Identifies AAC2′ as a Potential Drug Target with Naloxone Showing Potential Bait Drug Synergism

**DOI:** 10.3390/molecules27196150

**Published:** 2022-09-20

**Authors:** Vidya Niranjan, Akshay Uttarkar, Keerthana Murali, Swarna Niranjan, Jayalatha Gopal, Jitendra Kumar

**Affiliations:** 1 Department of Biotechnology, R. V. College of Engineering, Mysuru Road, Kengeri, Bangalore 560059, India; 2 Department of Artificial Intelligence and Machine Learning, R. V. College of Engineering, Mysuru Road, Kengeri, Bangalore 560059, India; 3 Department of Mathematics, R. V. College of Engineering, Mysuru Road, Kengeri, Bangalore 560059, India; 4 Bangalore Bioinnovation Centre, Helix Biotech Park, Electronic City Phase 1, Bangalore 560100, India

**Keywords:** *Mycobacterium tuberculosis*, time-series, genome analysis, AAC2′, drug repurposing, Metadynamics, drug synergism

## Abstract

The World Health Organization has put drug resistance in tuberculosis on its list of significant threats, with a critical emphasis on resolving the genetic differences in Mycobacterium tuberculosis. This provides an opportunity for a better understanding of the evolutionary progression leading to anti-microbial resistance. Anti-microbial resistance has a great impact on the economic stability of the global healthcare sector. We performed a timeline genomic analysis from 2003 to 2021 of 578 *mycobacterium* genomes to understand the pattern underlying genomic variations. Potential drug targets based on functional annotation was subjected to pharmacophore-based screening of FDA-approved phyto-actives. Reaction search, MD simulations, and metadynamics studies were performed. A total of 4,76,063 mutations with a transition/transversion ratio of 0.448 was observed. The top 10 proteins with the least number of mutations were high-confidence drug targets. Aminoglycoside 2′-N-acetyltransferase protein (AAC2′), conferring resistance to aminoglycosides, was shortlisted as a potential drug target based on its function and role in bait drug synergism. Gentamicin-AAC2′ binding pose was used as a pharmacophore template to screen 10,570 phyto-actives. A total of 66 potential hits were docked to obtain naloxone as a lead—active with a docking score of −6.317. Naloxone is an FDA-approved drug that rapidly reverses opioid overdose. This is a classic case of a repurposed phyto-active. Naloxone consists of an amine group, but the addition of the acetyl group is unfavorable, with a reaction energy of 612.248 kcal/mol. With gentamicin as a positive control, molecular dynamic simulation studies were performed for 200 ns to check the stability of binding. Metadynamics-based studies were carried out to compare unbinding energy with gentamicin. The unbinding energies were found to be −68 and −74 kcal/mol for naloxone and gentamycin, respectively. This study identifies naloxone as a potential drug candidate for a bait drug synergistic approach against *Mycobacterium tuberculosis.*

## 1. Introduction

In the year 1882, a German microbiologist named Robert Koch identified *Mycobacterium tuberculosis* (MTb) as the causative organism for tuberculosis (TB) [1]. The disease usually affects the lungs, leading to chest pains, fever, and persistent coughing [2]. The brief advice given to all TB patients in the 1800s was, “Just sleep and eat nutritious food”. In the year 2008, about 12,904 cases were reported at an incidence rate of 4.2 per 10,000 [1].

This discovery, combined with the subsequent discoveries of tuberculin in 1890, the Bacillus Calmette-Guerin (BCG) vaccine in 1908, and antituberculosis medications in 1943, gave a promise of the eradication of a disease that was deadlier than the plague. From the early to mid-twentieth century, mortality rates due to TB considerably decreased; nevertheless, research funding diminished, and medical and vaccine development stalled between 1970 and 1990. TB rates increased once more with the start of the AIDS pandemic and increase in resistant strains, as did interest in TB research and prevention [3].

In 2004, tuberculosis (TB) was responsible for 2.5 percent of all deaths worldwide. Extensive drug resistance (XDR), or multidrug resistance (MDR) plus resistance to second-line medications, has been a global concern in recent years, with XDR cases accounting for around 24% of all MDR cases in Russia in 2006 and about 9% of MDR cases worldwide in 2013 [4].

There were around 8.7 million new cases and 1.4 million deaths due to tuberculosis in 2011, with approximately two billion persons latently infected [5,6].

MDR-TB is a type of tuberculosis caused by bacteria that are resistant to the two most effective first-line anti-TB medications, isoniazid and rifampin. Multidrug resistant tuberculosis (MDR-TB) can be treated and cured with second-line medicines. MDR-TB is still a public health concern and security threat. In 2020, only around one-third of patients with drug-resistant tuberculosis received treatment [7].

*Mycobacterium tuberculosis* can be found in lung cavities, empyema pus, or solid caseous material, where antibiotic penetration is difficult, or the pH is low enough to prevent most antibiotics from working [8].

As isoniazid is primarily active against organisms developing aerobically in lung cavities, it is crucial early on in therapy; its bactericidal activity rapidly reduces the sputum viable count [9]. Pyrazinamide is exclusively active at a low pH, making it suited for destroying organisms inside necrotic foci in caseous tissue. This explains why pyrazinamide appears to be ineffective after the second month of treatment [10].

Rifampin resistance is caused by mutations in the rpoB gene, which encodes the beta subunit of RNA polymerase. The bulk of equivalent mutations in rpoB occur in a narrow area of fewer than 100 kb, with less than 5% happening elsewhere [11].

Streptomycin resistance is caused by mutations in the RrS and rpsL genes, which result in a change in the streptomycin binding site; however, these changes are only found in about half of the strains investigated [12].

Isoniazid resistance is a good example. Modifications to KatG, such as partial or whole deletions, point mutations, or insertions, result in the loss or reduction of catalase function, as well as high-level isoniazid resistance [13]. The pyrazinamidase gene is usually mutated in pyrazinamide-resistant organisms; however, the gene can be inactivated by inserting IS6110 [14].

All antituberculosis drugs have different rates of resistance emergence, with ethambutol having the greatest rate and rifampin and quinolones having the lowest [15].

To reduce the spread of drug-resistant strains and avoid the formation of multidrug-resistant strains, early detection of drug-resistant tuberculosis and judicious use of second-line treatments are recommended. Finally, if drug susceptibility tests are unavailable or results are delayed, physicians should recognize that patients who do not respond to empirical short-course chemotherapy that is directly observed are at high risk of developing multidrug-resistant tuberculosis and should be treated accordingly [16].

Although *Mycobacterium tuberculosis* has a modest genetic diversity compared with other pathogenic bacteria, the strain’s genetic background has been shown to influence several aspects of drug resistance evolution. The fitness costs and rate of resistance evolution of drug resistance mutations may differ depending on the genetic background. Treatment success rates for M. tuberculosis infections caused by MDR/XDR variations are frighteningly low, with just 54% of MDR and 28% of XDR cases [17].

There has been a race to create a better vaccination for the past ten years, and the Modified-Vaccinia-Ankara (MVA) 85A vaccine was introduced. Unfortunately, the optimism surrounding MVA85A clinical trials was dashed when the vaccine failed to increase TB protection [18].

As most patients with tuberculosis have a latent infection, new diagnostic and screening technologies and guidelines are needed to control the illness [19]. There are modern techniques for identifying single nucleotide polymorphism/s (SNPs) and mutational patterns [20]. In the proposed study, we provide an intense review of the mutations (SNPs, Indels, and deletions) from 2003 to 2021. This provides an overview of the extent of genomic variability and regions of low mutation. This leads to the identification of novel drug targets, along with a challenge to perform functional annotation of unexplored genes

## 2. Results and Discussion

### 2.1. Genomic Analysis of Mycobacterium Tuberculosis Provides Insights into Mutation Patterns

**Figure 1:**
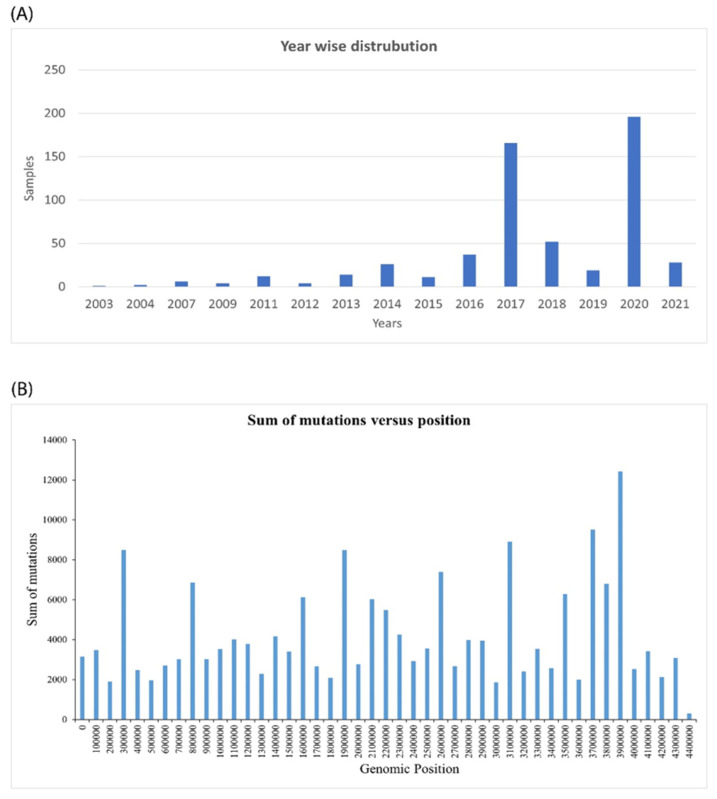
*Mycobacterium* time series analysis. (**A**) Year-wise distribution of samples collected from the NCBI database. (**B**) The mutations of *Mycobacterium tuberculosis* over the time series can be cumulatively plotted versus the genomic positions.

Over the course of 18 years, 578 assembled samples were subjected to genome analysis (2003–2021). The sample distribution for each year is tabulated in Figure 1A. The assembly name, assembly biosample accession, and strain characteristics for the 578 samples are included in Supplementary Table S1.

iVar was used to annotate the bam files, and the output was saved in vcf format. SNPeff was used to process the vcf files to capture the effect on protein function [21].

A total of 476,063 variants were detected across all 578 samples. The frequency of variance was determined to be one in every nine nucleotides. There are 430,660 single nucleotide polymorphisms (SNPs), 45,211 multiple nucleotide polymorphisms (MNPs), 147 inserts, 4 deletions, and 31 mixed variants among the overall number of variations. There were no inversions or duplications discovered.

A detailed count and percentage contribution of each category of variation are tabulated in Table 1.

From Table 1, upstream and downstream gene variants corresponded to 44.8% and 44.9% of mutations, respectively. The role of these mutations is a modifier in nature and has a low impact on protein function. Frameshift variations, start lost, and stop lost corresponded to 125, 818, and 1444 mutations, respectively. These variations have a high impact on protein function. Synonymous variants numbered at 90,276, which do not affect the protein. To visualize the mutations in a broader picture, we can conclude that missense mutations account for 290,087 or 75% of total mutations occurring in *Mycobacterium tuberculosis.* Nonsense and silent mutations were found to number 6247 with a ratio of 3.21.

The regions with the most variations are graphically represented in Figure 1B.

Classification of variations based on impact was as follows: high impact variations numbered at 9563, corresponding to 0.2% contribution. A total of 327,310 mutations were moderate in nature, contributing to 6.9%. Similarly, low-impact mutations were numbered at 90,853, contributing to 1.93%. The highest number of variations with counts of 4,253,865 accounted for modifiers contributing to 90.8% of total mutations.

As high-impact mutations have been of significance concerning *Mycobacterium tuberculosis* protein function and evolution over the years, a list of the top 10 proteins is provided in Table 2. This includes a sorted list of protein names along with gene ID, number of mutations, protein function, and pathway involved respectively. The complete list of all proteins with variations is available in Supplementary Table S2.

One of the major observations from the list of proteins is that these are either existing drug targets with or without gained microbial resistance via mutations, along with prospective targets. These findings suggest analyzing the regions with no variations or regions of less variability over a period of time.

### 2.2. 369 Genes with Unknown and Hypothetical Functions Were Annotated for Better Functional Insights and Target Selection

From the SNP analysis carried out in the study, a total of 1645 genes were found, all of which had no high-impact mutations. Out of these, 369 mutations were found to be with functions either hypothetical or unknown. The functional protein annotation analysis was initially carried out using two forked approaches. One with InterPro scan and the other with NCBI blast (National Centre for Biotechnology Information), followed by GO mapping, and merging the results from both the approaches to obtain a comprehensive annotation result.

From the InterPro scan, there were 312 sequences with IPS, 57 without IPS, and 88 without GOs.

Out of the 369 genes, 2 genes did not confer any blast results, and 133 genes provided results for blast only. Forty genes were mapped after the blast but could not be annotated. Finally, 194 genes were annotated following blast and mapping to the *Mycobacterium tuberculosis* genome.

Detailed information on mapped and annotated genes has been provided in Supplementary Table S3.

On completion of the functional annotation and pre-existing literature on the protein function of 1645 genes with zero high-impact mutations, they have been classified into major functional categories and tabulated in Table 3.

From the above, the mentioned protein function classes, “Virulence, detoxification, and adaption” was found to be of research interest to identify potential novel and prospective protein receptors as drug targets.

Amongst the 37 proteins, Aminoglycoside 2′-N-acetyltransferase (AAC) (Uniport ID: P9WQG9) [22] was selected for its features as a potential drug target.

### 2.3. Aminoglycoside 2′-N-Acetyltransferase (AAC2′): Mode of Action toward the Resistance of Aminoglycosides

The four families of aminoglycoside acetyltransferases are AAC(1′), AAC(2′), AAC(3′), and AAC(6′), which are named by the position of modification on the 2-deoxystreptamine core [23]. A gentamicin 2′-N-acetyltransferase, one of two aminoglycoside resistance factors in *Providencia stuartii*, is chromosomally encoded [24].

Several aminoglycosides, including gentamicin, tobramycin, dibekacin, kanamycin, and netilmicin, are modified by AAC(2′) enzymes found in gram-negative bacteria and Mycobacterium. There is just one subclass, which contains AAC(2′)-Ia (*Providencia stuartii*), AAC(2′)-Ib (*Mycobacterium fortuitum* and *Acinetobacter baumannii*), AAC(2′)-Ic (*Mycobacterium tuberculosis* and *Mycobacterium bovis*), AAC(2′)-Id (*Mycobacterium smegmatis*), and a putative AAC [25].

AAC(2′)-Ia transfers acetate to peptidoglycan from an integral membrane protein that transports it from cytoplasmic acetyl-CoA pools to the cytoplasmic membrane’s outer surface [26].

The aminoglycoside 2′-N-acetyltransferase enzyme encoded by the AAC(2′)-Ib gene acetylates the 2′-amino group of the aminoglycosides gentamicin, tobramycin, kanamycin B, netilmicin, and 6′-N-ethylnetilmicin [27].

Aminoglycoside acetyltransferases (AACs) catalyze the synthesis of a physiologically stable amide with the aminoglycoside using intracellular acetyl-CoA as a co-substrate. O-acetylation occurs using the acetyltransferase domain of the bifunctional enzyme AAC(6′)-APH(2′′) and the mycobacterial enzyme AAC(2′)-Ic [28], although AACs primarily change amino groups (N-acetylation).

The GCN5-related N-acetyltransferase (GNAT) class of proteins includes AACs. X-ray crystal structures of four enzymes, (AAC(6′Ii), AAC(3)-Ia, AAC(2′)-Ic, and AAC(6′)-Iy), show that the aminoglycoside binding pocket commonly contains a highly negatively charged surface to accommodate the polycationic antibiotic, even though none of the enzymes in this class have significant primary sequence homology or conserved catalytic residues.

AACs are further categorized based on the aminoglycoside structure acetylation site. The point along the amino sugar/aminocyclitol targeted is stated in brackets by convention, and the amino sugar/aminocyclitol change is marked in brackets after the attack position [29,30].

**Figure 2:**
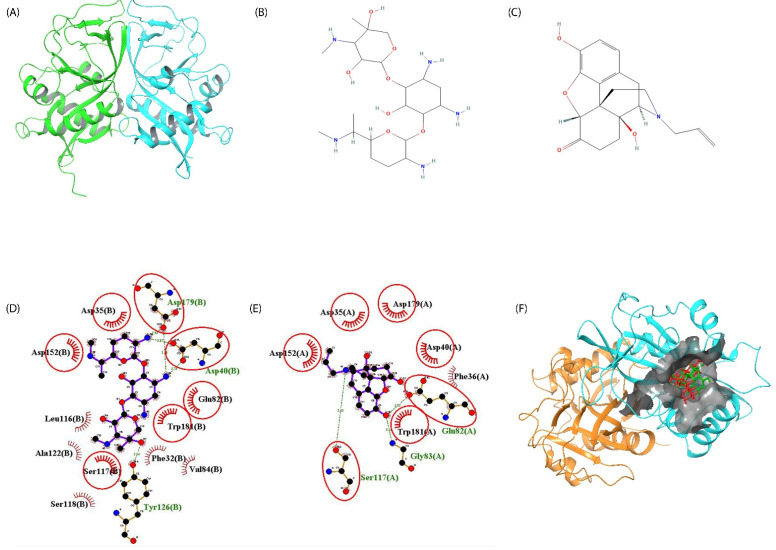
Comparison of 2D interaction profile. (**A**) The tertiary structure of AAC2′ retrieved for protein data bank (PDB ID: 1M4I) with chain A colored in green and chain B colored in cyan. (**B**) Fischer projection of gentamicin. (**C**) Fischer projection of naloxone. (**D**) The surface visualization of the binding pocket in AAC2′ is shown as a gray patch on which both gentamicin (red) and naloxone (green) compounds can be visualized, binding to the same region. The images were developed using Maestro v13.2 (Schrodinger 2022-2). (**E**) The 2D interaction profile of gentamicin with the interactive residues of AAC2′ binding site. (**F**) The 2D interaction profile of naloxone with the interactive residues of AAC2′ binding site. For C and D, the residues having hydrogen bonds are showcased in atomic representations with the bond length specified in angstrom. The hydrophobic interactions are shown in the residue name with a beaming red semicircle. The common residues in both interactions are encircled in red. The plot was developed using Ligplot+.

Performing the AAC2′ surface analysis, it can be sorted into 66 patches based on the criteria of positive, negative, and hydrophobic nature of the residues. AAC2′tertiary structure is provided in Figure 2A.

The total positive surface area is 8246.12 Å^2^ and the total negative surface area is 5211.96 Å^2^. The sum of donor surface area is 2753 Å^2^ and acceptor surface area is 3253 Å^2^. The overall charge of the protein in its native state is −3e.

The substrate and co-enzyme binding site is a positive patch with an area of 1326.91 Å^2^. The patch is constituted of 40 residues, of which there were 10 arginine, tryptophan, and tyrosine residues each. No cysteine residues were found. No disulfide linkages were found. Gln26 is found to be a deamination site and oxidation sites are reported as follows Trp90, Met1, His49, His2, His55, and Tyr80. No glycosylation and proteolysis sites were found.

Detailed information on each of the patches is provided in Supplementary Table S4.

### 2.4. Screening of Phyto-Actives as Potential Drug Candidates with Potential Features as Aminoglycosides

Initially, Set 2 compounds encompassing the eight most commonly used aminoglycosides were subjected to molecular docking with AAC2′. The docking results were sorted based on the docking score in descending order of priority. The best-docked aminoglycoside was gentamicin with a score of −6.036.

The Fischer projection of gentamicin is provided in Figure 2B.

The interacting amino acids are Asp40, Glu82, Asp179, and Trp189. Hydrogen bonds, salt bridge mediation interactions, and metal coordination were observed. The major components involved were donor atoms, acceptor atoms, and positive ionic atom features. Gentamycin, in its binding interaction with AAC2′, had O7 and O11 atoms that were donors in nature imparting energy of −1.19 kcal/mol and −1.60 kcal/mol, respectively, toward stable binding. O8 was acting as an acceptor atom with an energy of −0.42 kcal/mol. As suggested by previous research, interaction of nitrogen in the binding and acetylation of aminoglycosides is critical. The same observations were found as N12 of gentamycin was acting as a positive ionic atom, contributing energy of −2.0 kcal/mol.

These recorded features were used as a template to screen the phyto-active database from MolPort, consisting of 10,570 compounds (Set 1 compounds, as mentioned in Methods). For the threshold of feature match set to 4/4, a total of 66 compounds were matched. These compounds were further subjected to molecular docking with the AAC2′ receptor. The compounds were filtered based on score, with a docking score greater than gentamicin. A total of four compounds remained, of which naloxone was an FDA-approved drug that has the potential to bind with AAC2′, imparting a similar effect and with similar binding sites as gentamicin. A Fischer projection of naloxone is provided in Figure 2C.

For the docking studies, in spite of kanamycin being co-crystallized, we performed docking with eight aminoglycosides as part of site-specific docking with the site set to residues bound to co-crystallized kanamycin. The docking score showed gentamicin with a docking score better than kanamycin, hence gentamicin was selected as a “control” for the docking studies to develop an interaction template for the screening of phyto-actives. Apart from this, blind docking was performed to verify the selection of phyto-actives. Naloxone was the best molecule that bound to the same site in both site-specific docking and blind docking. This provides crucial cross-validation for the selection of naloxone. The ligand binding RMSD has been observed to be as follows: naloxone–kanamycin with 4.64 Å and naloxone-gentamicin with 4.63 Å.

The complete list of compounds with their structure, name, and docking score is provided in Supplementary Table S5.

Gentamicin forms a hydrogen bond with Asp40, Tyr126, and Asp179, whereas naloxone forms hydrogen bonds with Glu82, Gly83, and Ser117. The detailed interaction is available in Table 4. The AAC2′–gentamycin interaction profile can be seen in Figure 2E and the AAC2′–naloxone interaction profile in Figure 2F, respectively.

### 2.5. Naloxone–AAC2′ Interaction Profile as a Bait Drug Synergistic Effect with Aminoglycosides

Naloxone and gentamicin were the compound of interest and positive control for the In-silico analysis, respectively. In principle, a huge dataset of phyto-actives was screened and naloxone was shortlisted to effectively act as gentamicin, but not to undergo acetylation. Both the compounds bind the conserved substrate-binding amino acids 81–82 and 151–152. The binding pose of gentamicin and naloxone in the common binding pocket can be visualized in Figure 2F. The grey region on the binding surface with gentamicin is highlighted in red and naloxone in green.

The residues encircled in red highlight the common residues in both interactions. The plots were developed using Ligplot+ [31].

Both gentamicin and naloxone receptor complexes were subjected to molecular dynamics studies (MDS) for 200 ns. In previous studies, MDS for longer durations were shown to be crucial in gaining better insight into the interaction profile. MDS has also been shown to provide better insight into drug interactions [32,33,34,35].

**Figure 3:**
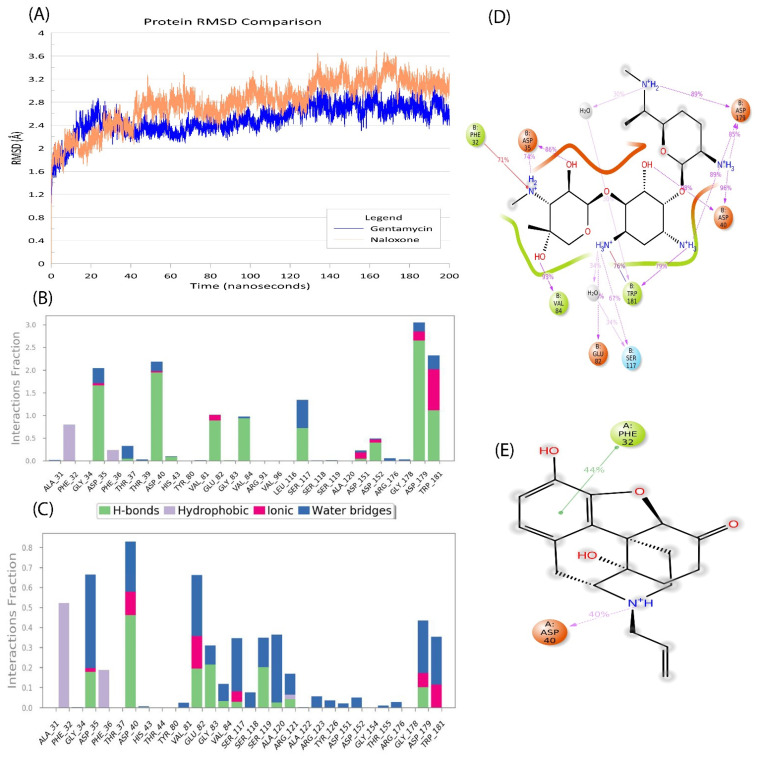
Molecular dynamics simulation profile for 200 ns. (**A**) A root mean square deviation (RMSD) profile of gentamicin (blue) and naloxone (light orange) has been plotted against time. The time of 200 ns is shown as 10,000 frames on the x-axis and RMSD change on the y-axis. (**B**) The protein–ligand contacts and type of interaction for gentamicin occurring over the simulation period are shown. (**C**) The protein–ligand contacts and type of interaction for naloxone occurring over the simulation period are shown. For both B and C, H-bonds are green, hydrophobic interactions are light purple, ionic interactions are pink, and water bridges are blue. The stacked bar charts are normalized over the course of the trajectory; for example, a value of 0.7 indicates that the specific interaction is maintained for 70% of the simulation time. As some protein residues may have several interactions of the same subtype with the ligand, values above 1.0 are feasible. (**D**) The 2D profile of gentamicin interactions after the simulation period with the residues in the binding sites (**E**) The 2D profile of naloxone interactions after the simulation period with the residues in the binding sites. For both D and E, the color codes signify the residues in orange are negatively charged, green are hydrophobic, and light blue are polar in nature. The interactions shown in pink are pi stacking and the ones in green are hydrogen bonds. The images were developed using Maestro v13.2 (Schrodinger 2022-2).

One of the most important parameters to consider when analyzing MDS data is the protein root mean square deviation (RMSD). RMSD is a metric for calculating the average change in displacement of a group of atoms in relation to a reference frame. Gentamicin–AAC2′ had a protein RMSD of ~2.6 Å in comparison with ~3.0 Å induced by naloxone to the AAC2′ protein. The plot of this is available in Figure 3A.

In both simulations, during the simulation period of 200 ns (10,000 frames), the fluctuations were stable with no major deviations.

Similarly, ligand RMSD can provide crucial insight into the movement of the ligand within the binding pocket. Gentamicin had a ligand RMSD value of ~0.9 Å and naloxone of ~0.75 Å. The subtle changes in ligand RMSD suggest that naloxone is more tightly bound to AAC2′ with less movement in the binding pocket. Similar observations were also made in the radius of gyration analysis with naloxone at ~3.21 Å and gentamicin at ~4.4 Å. Both the above-mentioned variables suggest that naloxone-AAC2′ binding is more “fit” than the gentamicin-AAC2′ complex.

Understanding the changes in bonds created and dissociated in the complex requires an understanding of protein—ligand interactions. The exchanges that took place were divided into four categories. In ligand binding, hydrogen bonds (H-bonds) are important. Due to their considerable influence on drug selectivity, metabolization, and adsorption, hydrogen-bonding properties should be considered in drug design. The four forms of hydrogen bonds that exist between a protein and ligand are backbone acceptors, backbone donors, side-chain acceptors, and side-chain donors.

For the gentamicin-AAC2′ complex, exclusive hydrogen bonding occurred with no amino residues, and the same was observed in the naloxone-AAC2′ complex. However, various residues have shown hydrogen bonding mediated via water bridges. Water bridges are connections mediated by a water molecule between hydrogen-bonded proteins and their ligands. This hydrogen-bond geometry differs slightly from the standard H-bond definition.

Mediated h-bonds for gentamicin-AAC2′ was observed in Gly34, Trp39, Val81, Gly83, Leu116, Asp179, and Trp181. Similarly, naloxone-AAC2′ showed interactions at residual positions Gly34, Thr37, and Val81. The above-mentioned interactions were found for >50% of the simulation period. In both cases, the conserved residue Val81, which is an important part of the aminoglycoside binding pocket, was found. Amongst these interactions, ionic interactions also play a role. Ionic or polar interactions occur when two oppositely charged atoms come within 3.7 Å of one other and there is no hydrogen bond between them. We also track protein—metal—ligand interactions, which were defined by a metal ion coordinated within 3.4 Å of the heavy atoms of both the protein and ligand (except carbon). The presence of ionic interactions is due to either N^+^H_3_ and N^+^H_2_ atoms. Finally, hydrophobic interactions were observed at Phe32 for both the gentamicin-AAC2′ complex and naloxone-AAC2′ complex. Generally, these types of interactions involve a hydrophobic amino acid and an aromatic or aliphatic group on the ligand. The bar plot of protein–ligand contacts with respect to simulation time for gentamicin is provided in Figure 3B. Similarly, naloxone is provided in Figure 3C.

The 2D interactions of gentamicin with residues in the binding pocket after 200 ns simulations are provided in Figure 3D and naloxone is provided in Figure 3E.

The energy necessary for unbinding gentamicin and naloxone from the binding pocket of the AAC2′ receptor, designated as dissociation free energy (DFE), was investigated using Metadynamics. This method has been utilized to compute the complex free energy [36] and ligand binding free energy [37] in previous investigations. Metadynamics is a method of modifying the potential of one or more chosen variables (“collective variables”) by periodically adding a repulsive potential of Gaussian form at a position determined by the variable values. These repulsive Gaussians gradually fill up the sampled well, forcing the calculation to sample somewhere else. The sum of the Gaussians and the free-energy surface (FES) becomes flat at particular points in the simulation, and so the sum of Gaussians is the negative image of the FES.

The in silico analysis is usually carried out on multiple runs of 20 to 50 ns each. In the present study, 10 simulation runs were carried out for 50 ns, with changes made in random seed numbers from 2007 to 2016 for both complexes. The values obtained for each run was plotted. The DFE obtained from each of the runs was also plotted. If convergence was not achieved, the best fit line was plotted to obtain the final DFE. For both complexes, simulations were found to be two-way runs in nature. The ligand, after unbinding from the binding pocket again, returns to the binding pocket. This suggests that the currently docked pocket is the best region for the ligands to bind. They tend to freely move around the receptor for stable interactions and return to the binding pocket. This is observed throughout the simulation period.

**Figure 4:**
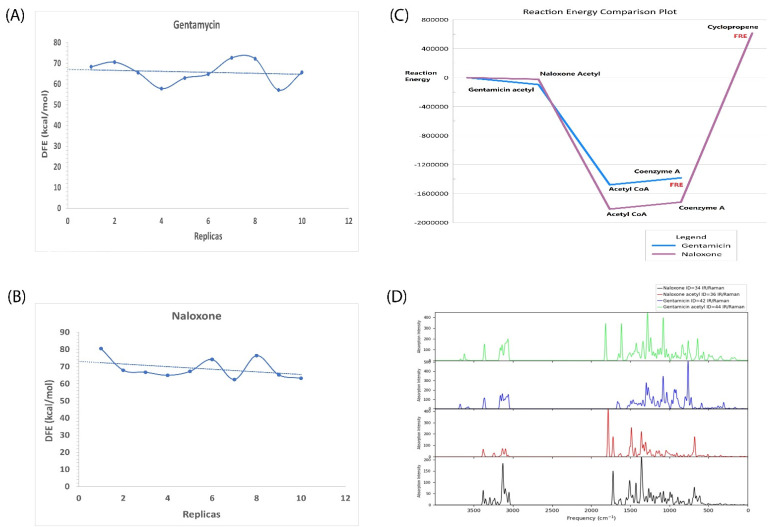
Reaction energies and unbinding energy profile. (**A**) The dissociation free energy (DFE) values of gentamicin for each replica simulation study are shown. (**B**) The dissociation free energy (DFE) values of naloxone for each replica simulation study are shown. The trendline is shown in a dotted blue line. (**C**) The reaction energy profile of each of the reactants and products formed is plotted with in-text mentions for each of them. The final reaction energy (FRE) is in red. The reaction profile of gentamicin is highlighted in dark purple and naloxone in blue. The x-axis consists of energy values in cal/mol. (**D**) The IR spectra of naloxone, naloxone acetyl, gentamicin, and gentamicin acetyl are shown in black, red, blue, and green, respectively. The x-axis denotes frequency/cm with absorption intensity in the y-axis.

The DFE for gentamicin was found to be −68 kcal/mol and for naloxone at −74 kcal/mol. The results suggest naloxone to be in a better-bound state than gentamicin. The DFE values obtained for each of the replicas are provided for gentamicin in Figure 4A and naloxone in Figure 4B.

### 2.6. In Silico Validation of Naloxone Acetylation to Be Highly Unstable and Disordered

Acetylation of aminoglycosides occurs via transferring of the acetyl group to the 2′ amino group of the aminoglycoside. In the current study, acetylation of gentamicin in the presence of Acetyl-CoA is referred to as Reaction 1 and the hypothesized naloxone acetylation is referred to as Reaction 2.

A brief stoichiometry reaction is shown below (Reactions (1) and (2))

(1) $$\mathrm{Gentamicin}\;(C_{21}H_{43}N_{5}O_{7})+\;\mathrm{Acetyl}-\mathrm{CoA}\;(C_{23}H_{38}N_{7}O_{17}P_{3}S)\;\to \mathrm{Acetylated}\;-\mathrm{gentamicin}\;(C_{23}H_{45}N_{5}O_{8})\;+\;\mathrm{CoA}\;(C_{21}H_{36}N_{7}O_{17}P_{3}S)$$

(2) $$\mathrm{Naloxone}\;(C_{19}H_{21}NO_{4})\;+\;\mathrm{Acetyl}\;-\;\mathrm{CoA}\;(C_{23}H_{38}N_{7}O_{17}P_{3}S)\;\to \;\mathrm{Acetylated}\;-\;\mathrm{Naloxone}\;(C_{18}H_{19}NO_{5})\;\;+\;\mathrm{CoA}\;(C_{21}H_{36}N_{7}O_{17}P_{3}S)\;+\;\mathrm{Cyclopropene}\;(C_{3}H_{4})$$

From the above reactions, it can be observed that Reaction (1) is conserved in nature and no additional atoms or by-products are required. However, for Reaction (2) to be stoichiometrically possible, cyclopropene should be produced as a by-product of the reaction. This aspect of the reaction is crucial as it is difficult to produce and highly reactive.

Reaction (1) has a final reaction energy (FRE) of −1383.54734 kcal/mol and Reaction (2) has a final energy reaction of 612.24846 kcal/mol. Due to the stoichiometric requirement of cyclopropene as a by-product, Reaction (2) is thermodynamically unfeasible. This can be concluded as a chemical and thermodynamic validation that acetylation of naloxone is not possible under natural conditions.

Further, we analyzed and provided a comparison of the heat of formation of acetylated gentamicin and acetylated naloxone. At 0 K, the former has a ΔH of formation of −325.4 kcal/mol and the latter has a ΔH of formation of −164.71 kcal/mol. At 298K, ΔH of formation for gentamicin and naloxone is −366.38 and −182.01 kcal/mol, respectively.

The entropy values at 298.15 K and 1.00 × 10^0^ atm required to change gentamicin to the acetylated form of gentamicin is 168.34 to 169.56 cal/mol/K, with an increase of 1.22 cal/mol/K. Similarly, to change naloxone to the acetylated form of naloxone, the entropy values change from 128.28 to 129.13 cal/mol/K, with an increase of 0.84 (cal/mol/K). Evidently, gentamicin acetylation is a more favorable reaction than naloxone acetylation.

The reaction energy comparison plot is provided in Figure 4C. An In-silico IR/Raman spectroscopy plot is provided in Figure 4D. The comparison with experimental spectra ensures the exact chemical reactions.

## 3. Discussion

The two primary classes of aminoglycoside-modifying enzymes in Mycobacteria are acetyltransferase and phosphotransferase [38].

N-acetyltransferase was discovered in the *Mycobacterium tuberculosis* genome, and aminoglycoside N-acetyltransferase (AAC2′), the best biochemically characterized aminoglycoside-modifying enzyme, can acetylate all known aminoglycosides with a 2′ amino group, including neomycin, ribostamycin, kanamycin, gentamycin, and tobramycin [39].

In addition to drug target alteration, which can inhibit antibiotic binding to the target site, Mycobacterium tuberculosis develops intrinsic drug resistance to significant antibiotics. These target modifications in *Mycobacterium tuberculosis* limit the binding of antibiotics such as lincosamides, macrolides, and streptomycin [40,41].

In Section 2.3, an elaborate mode of aminoglycoside interaction with AAC2′ is discussed, with gentamicin as a template for the screening of phyto-actives. Mycobacterium tuberculosis is a major threat due to its development anti-microbial resistance. The major focus of the current experiment was to deliver a novel drug candidate to counter drug resistance. In this regard, a time series genome analysis was performed to capture predominant sites of mutations and corresponding proteins. This led to the identification of proteins that have not undergone any variation over the years. AAC2′ is a crucial protein in the scavenging and elimination of aminoglycosides. This is a key defense mechanism, and its mode of action is resistance to antibiotics. With the help of feature screening and molecular docking studies, naloxone was shortlisted as a potential FDA-approved natural derivative drug for re-purposing. Gentamicin (aminoglycoside) was used as a positive control for comparison studies against naloxone. In silico validation suggested that acetylation of naloxone was not naturally thermodynamically possible. It had a better binding, interaction, and unbinding profile compared with gentamicin. In this regard, naloxone could be used as a bait drug to competitively bind at the aminoglycoside site of binding, providing an opportunity for aminoglycosides to avoid AAC2′ scavenging.

Due to the uncanny ability of mycobacteria to mutate and adapt toward drug resistance, any variation induced in AAC2′ will lead to a loss of function, either in its ability to bind to CoA or aminoglycosides. This will, in turn, be beneficial to the activity of aminoglycosides. In the future, we hope to validate these findings in vitro and repurpose naloxone as an anti-TB drug.

## 4. Materials and Methods

### 4.1. Retrieving the Data from Public Datasets

For the current study, 578 assembled whole genome datasets from 2003 to 2021 were downloaded from NCBI datasets for *Mycobacterium tuberculosis*. The genomic sequence was downloaded in fasta format for analysis. The reference genome considered in the study is Mycobacterium tuberculosis H37Rv, bearing NCBI reference number NC_000962 [20].

### 4.2. Mapping of Samples with Reference Genomes and Variant Calling

BBMap [42] is a splice-aware global aligner for DNA and RNA sequencing reads. The read input for BBMap must be fasta or fastq, compressed or raw. Long RNA-sequence reads [43] and BB merge [44] have both been utilized with this technique. A detailed description of the dependencies, installation, and running of the package is available at the URL in the respective reference.

The tools generated the coverage information using pileup. This avoided the need to use mpileup before variant calling. The output file was stored in bam format for further processing.

#### Variants Calling

iVar [45] is a computational package for detecting the variants of reference-aligned genomic sequences (in bam format). A detailed description of the dependencies, installation, and running of the package is available at the URL mentioned in the respective reference.

The tool is also available at Galaxy Webserver [46] with a user-friendly interface for the uploading of bam files and running of iVar analysis pipeline. The output was stored in vcf format for further processing.

### 4.3. Annotation of Variants

The genetic variations and their functional effects were analyzed from the vcf files using SNPEff [47]. SNPEff needs a database to perform genomic annotations. This feature is also available in Galaxy Webserver for analysis. There are pre-built databases for thousands of genomes. As the *Mycobacterium tuberculosis* database was not available in the pre-built database, it was built using the reference genome used for alignment. Details related to configuring a genome and building a genome from various sources are available in the respective reference via the URL.

The annotation was performed with default splicing regions. The output files were saved as annotated vcf and HTML file formats.

### 4.4. Functional Annotation

(a) Downloading the Genomic Sequences

The results from the SNP analysis promoted our research interest to shortlist the genes and proteins with no mutations consistently across the time series for the *Mycobacterium tuberculosis* genome. A total of 1645 genes was used, for which corresponding protein sequences were downloaded from the NCBI dataset in fasta format.

(b) InterPro Scan

The genomic sequences were uploaded to the EMBL InterPro [48,49] scan web server. The following databases were selected for analysis: (a) TIGRFAMs, (b) SFLD, (c) PANTHER, (d) HAMAP, (e) PROSITE profiles, (f) PROSITE patterns, (g) SMART, (h) CDD, (i) PRINTS, (j) Pfam, and (k) PIRSF.

(c) NCBI BLAST Search

NCBI BLAST [50] is an open-source web server to find sequence-based matches to protein sequences in the NCBI database. We used the Blastx program against the non-redundant protein sequences database (nr v5). The BLAST Expect value (E-value) was set to 1.0 × 10^−3^ for optimal robust query hits. The number of hits to be returned was set to 20. Under the blast parameters, word size was set to 6. The HSP length cut-off was set to 33, along with HSP-hit coverage set to 0, as a part of filtering the results. The optimal parameters ensured that results would have high confidence and no false positives.

(d) GO Mapping and Annotations

Blast2GO v6.0 [51] is a state of the art functional analysis tool available on all platforms, and its basic version is free for users. The following parameters were set for annotation: the Annotation cut-off was set to 55 and GO weight to 5. The E-value hit filter was set to 1.0 × 10^−3^ and the number of hits to be returned was set to 500.

(e) Merge Results from InterPro and GO Annotations

The GO annotations from the InterPro scan were added to the output of GO annotations from Blast2GO. The results were compiled, and graphical presentations were made using open-source software.

(f) Functional Clustering of Genes

The functional analysis provided classification for 369 proteins for which the existing function from the literature was either unknown or set to be hypothetical in nature. Based on the role of such proteins, 10 categories were made based on the gene functions.

The complete functional annotation was carried out using OmicsBox 2.2 [52].

### 4.5. Molecular Docking

(a) Protein Selection and Preparation

Aminoglycoside 2′-N-acetyltransferase (AAC2′) (PDB: 1M4I) was selected for the docking studies as a protein receptor. In the Protein Data Bank, 4 structures of AAC2′ are available. All the 3D structures have similar characterization methods and amino acid coverage. The receptor 1M4I has the best resolution and has kanamycin co-crystallized along with the protein structure

Docking and virtual screening were performed using the Small Molecule Discovery Suite 2021-4. Other tools used included Protein Preparation Wizard [53], Epik [54], Impact, LigPrep, Prime [55], Receptor Grid Generation, and Glide [56,57].

To determine residues, HET atoms, and to validate valency, the protein was pre-processed. During pre-processing, bond ordering was assigned, and hydrogen atoms were replaced. Prime was utilized to compensate for the lack of side chains. Epik was used to construct het states with pH values of 7.4 ± 2.0. During structural refining, PROPKA [58] was utilized to assign hydrogen bonds for neutral pH (7.0). Structure minimization for less than 0.30 was achieved using the OPLS3e [59] force field. To find potential binding sites, researchers looked at the literature as well as at prebound kanamycin (substrate) sites from the Protein Data Bank.

For docking compounds with the selected binding pocket, a receptor grid was generated without any specified limitations or excluded volumes. No pre-atom scale factors were set during grid generation.

The docking was carried out in two modes, wherein: 1. Blind docking with receptor grid was generated for the whole protein; 2. Site-specific docking with the grid was generated on sites specified for docking (substrate binding). The substrates in this case were aminoglycosides. A virtual screening workflow was set up to screen the phyto-actives. In the first stage, high-throughput virtual screening (HTVS) was flexibly carried out by penalizing non-polar conformations. After docking, post-docking minimization was carried out by retaining the top 10 percent of best-docked phyto-actives. All the scoring states were retained.

In the second stage, standard-precision docking (SP) was carried out for the retained phyto-actives with similar parameters as that of HTVS, but only the scoring states of the top 10 percent of phyto-actives carried forward were considered.

In the final stage, extra-precision docking was carried out using the same parameters as above and the best scoring states were taken as the final docking results [60,61,62].

(b) Ligand Preparation.

For the current study, two sets of ligands were considered for docking studies. Set 1 consisted of phyto-actives (natural source and naturally derived) from the MolPort database (https://www.molport.com/) (accessed on 12 August 2021). A total of 10,570 phyto-actives were imported for the docking studies. This set also consisted of 79 phyto-actives that were approved by The United States Food and Drug Administration (FDA) as drug candidates for various ailments.

Similarly, Set 2 consisted of standard aminoglycosides as a positive control to compare against the phyto-actives. These compounds were downloaded from PubChem [63]. Set 2 consisted of amikacin (PubChem CID: 37768) [64], gentamicin (PubChem CID: 3467) [65], kanamycin (PubChem CID: 6032) [66], neomycin (PubChem CID: 8378) [67], paromomycin (PubChem CID: 165580) [68], plazomicin (PubChem CID: 42613186) [69], streptomycin (PubChem CID: 19649) [70], and tobramycin (PubChem CID: 36294) [71].

The LigPrep module was used to prepare the ligands for the docking studies. The compounds under consideration were imported as either Set 1 or Set 2. Ionization was set to generate all possible states for a target pH of 7.0 ± 2.0 and to generate tautomers. Stereoisomers were generated by retaining specified chirality. The output structures were stored in SDF format.

### 4.6. Feasibility of Reaction Studies

Jaguar [72] is a module that performs ab initio quantum mechanics calculations for the electronic structure of molecules. In our current study, the reaction energetics for the acetylation of gentamicin and naloxone were calculated.

The reaction panel in Jaguar was used to set up the reaction energetics calculations. The reaction tab can be used to import and define the reactants and products in the chemical reaction. In the molecules tab, symmetry was set to use if present with charge and multiplicity was set to the molecular inherit property. The standard split valence double basic set, 6-31G [73], was used with polarization set to ** and diffuse to none.

The calculations were performed based on density function theory with the SCF spin treatment set to automatic to run a spin-unrestricted calculation for open-shell systems. For heavy metals, it is important to incorporate relativistic effects. Thus, a non-relativistic option was selected, as the molecules lacked heavy metals.

Under SCF settings, the scheme set to DIIS with the accuracy set to ultrafine for pseudo-spectral grids with a tight cut-off. The initial guess was set to atomic overlap as the geometry of molecules was not refined at an atomic level. As for the convergence, the SCF level shift was set to 0.0 for Hartree with no thermal smearing. For solvation, the Poisson-Boltzmann Finite (PBF) model was selected, as it produces better energies than other PCM models. Water was set as the solvent with gas phase reference to be selected from the optimized gas-phase structure.

The output files were written in Gaussian format.

Visualization of IR frequency spectra was plotted using the inbuilt “plot spectra” panel.

### 4.7. Molecular Dynamics Simulation Studies

Desmond [74] was used for performing simulations to confirm the stability and interaction mapping between AAC-naloxone and AAC-gentamycin (control). The interaction complex was subjected to protein pre-processing and H-bond assignment with similar parameters, as mentioned earlier. The simulation system was built utilizing the system builder. The solvent model selected was TIP3P [75], with boundary conditions defined by an orthorhombic box with minimized volume encapsulating the complex. The force field applied was OPLS3e. The system was neutralized by adding Na^+^ ions.

To perform the molecular dynamics simulation, the system was imported from the workspace. Simulation time was 200 ns, with the trajectory recording interval set to 0.1 ns (the optimized interval time helps in clear visualization and appropriate storage space). The ensemble class was set to NPT (which defines the thermodynamic parameters of constant pressure and temperature, a variable volume that allows the protein to undergo conformational change). The complete simulation protocol involved the following steps: 1. Simulate with Brownian dynamics NPT, T = 10 K, with small time steps and restraint on solute heavy atoms for 100 ps; 2. Simulate with Brownian dynamics NPT, T = 10 K with small time steps and restraint on solute heavy atoms for 12 ps to stabilize simulation system; 3. Solvate the pocket; 4. Simulate NPT with no restraints for 100 ps; 5. Run the final simulation for 100 ns with time step set to 2 femtoseconds (fs) and a temperature of 310 K. Use a cut-off short-range method with a radius of 9.0 Å (This optimized value avoids overlapping of atoms. Simulation has a temperature increase of 10 K per time step after the solvation of the binding pocket.

The long-range electrostatic interactions and cut-off were calculated using the particle mesh Ewald method. The simulation interaction diagram tool was used to analyze simulation results for change in RMSD and protein-ligand contacts for a detailed description of the steps involved please refer to the following papers [76,77].

### 4.8. Well-Tempered Metadynamics Simulation Studies

The Metadynamics [78] module of Desmond was used to carry out the analysis. The height and width of the Gaussian potential, as well as the interval at which the Gaussians are added, are the parameters that influence simulation accuracy. The height-to-interval ratio has a minor effect on accuracy; nonetheless, lesser values of this ratio slightly boost accuracy. During a free MD run, the Gaussian breadth should be about 1/4 to 1/3 of the average fluctuations of the collective variable.

A wall with a CV is equal to the sum of the biggest dimensions of the complex. The wall was set to 30 Å in this investigation, which contained the entire receptor–ligand complex.

The time interval between injections of a Gaussian was set to 0.09 picoseconds (ps). Temperature and pressure in the simulation were set to 310 K and 1.01325 bar, respectively. The simulation time was set to the amount of time we wanted the simulation to last (25–50 ns).

The dynamics of the system were effectively accelerated (without heating the system) up to T + T during well-tempered metadynamics, where T is the specified MD simulation temperature.

There are two limiting cases for ΔT (the bias factor kTemp):

ΔT → 0: The well-tempered metadynamics run is identical to a standard MD simulation at temperature T.

ΔT → ∞: At temperature T, the well-tempered metadynamics simulation is similar to a regular MD simulation.

The standard or non-well-tempered metadynamics run is the same as the well-tempered metadynamics run. The highest barrier in the simulation system that the well-tempered metadynamics run should surmount guides the choice of the bias factor value.

The detailed protocol is available at [79]. Calculation of dissociation free energy (DFE) from the free energy surface (FES) data is available at [80].

## Figures and Tables

**Table 1:** A detailed tabular presentation of types of variations occurring in *Mycobacterium tuberculosis*, along with its count and percentage.

Sl No.	Type of Variation	Percentage	Count
1	Conservative inframe insertion	0%	19
2	Disruptive inframe deletion	0%	1
3	Disruptive inframe insertion	0%	3
4	Downstream gene variant	44.91%	2,103,270
5	Frameshift variant	0.00%	125
6	Initiator codon variant	0.00%	183
7	Intergenic region	1.00%	46,659
8	Intragenic variant	0.09%	4113
9	Missense variant	6.99%	327,309
10	Splice region variant	0.03%	1560
11	Start lost	0.02%	818
12	Start retained variant	0%	13
13	Stop gained	0.15%	7177
14	Stop lost	0.03%	1444
15	Stop retained variant	0.00%	116
16	Synonymous variant	1.93%	90,276
17	Upstream gene variant	44.84%	2,099,823

**Table 2:** List of top 10 proteins with the highest number of mutations in each of the proteins accounting for high impact mutations.

	Protein Name	Gene ID	High Impact Mutations	Protein Function	Pathway
1	pks2	Mb3855c	46	Function unknown; supposedly involved in lipid metabolism.	Lipid metabolism
2	pks1	Mb2971c	45	Polyketide synthase possibly involved in lipid synthesis.	Lipid metabolism
3	Mb3933c	Mb3933c	40	Hypothetical alanine and proline-rich protein.	Conserved hypotheticals
4	mmpL8	Mb3853c	40	Thought to be involved in the transport of lipids; it is required in the production of a sulfated glycolipid, sulfolipid-1 (SL-1).	Cell wall and cell processes
5	pks13	Mb3830c	38	Involved in the final steps of mycolic acid biosynthesis. Catalyzes the condensation of two fatty acyl chains.	Lipid metabolism
6	gltB	Mb3889c	37	Probably involved in glutamate biosynthesis [catalytic activity: 2 L-glutamate + NADP(+) = L-glutamine + 2-oxoglutarate + NADPH].	Intermediary metabolism and respiration
7	pks12	Mb2074c	33	Involved in the biosynthesis of mannosyl-beta-1-phosphomycoketide (MPM).	Lipid metabolism
8	Mb3018	Mb3018	31	Unknown; COULB is involved in the efflux system (possibly drug).	Cell wall and cell processes
9	embA	Mb3823	30	Involved in the biosynthesis of the mycobacterial cell wall arabinan and resistance to ethambutol (EMB; Dextro-2,2′-(ethylenediimino)-DI-1-butanol). Polymerizes arabinose into the arabinan of arabinogalactan [catalytic activity: UDP-L-arabinose + indol-3-ylacetyl-Myo-inositol = UDP + indol-3-ylacetyl-myo-inositol L-arabinoside].	Cell wall and cell processes
10	glnE	Mb2245c	29	Regulatory protein is involved in the regulation of glutamine synthetase activity. Adenylation and deadenylation of glutamine synthetase. Possibly regulates GLNB|Rv2919c [catalytic activity: ATP + [L-glutamate:ammonia ligase (ADP-forming)] = pyrophosphate + adenylyl-[L-glutamate:ammonia ligase (ADP-forming)]].	Intermediary metabolism and respiration

**Table 3:** List of functional categories and number of proteins in each category.

Sl No.	Protein Function Category	Number of Proteins
1	Cell wall and cell processes	305
2	Conserved hypotheticals	588
3	Information pathways	35
4	Insertion seqs and phages	39
5	Intermediary metabolism and respiration	371
6	Lipid metabolism	105
7	PE/PPE	40
8	Regulatory proteins	87
9	Stable RNAs	34
10	Unknown	2
11	Virulence, detoxification, adaptation	37

**Table 4:** Protein-ligand hydrogen bond interaction profile.

Gentamycin	Asp40		Tyr126		Asp179	
Atom	Atom	Distance (Å)	Atoms	Distance (Å)	Atom	Distance (Å)
N2	OD1	2.74	OD2	2.99		
N4	OD2	2.87	OD2	2.53		
O7					OH	2.94
**Naloxone**	**Glu82**		**Gly83**		**Ser117**	
O1	OE1	2.62	N	3.12		
N1					OG	3.03

## Data Availability

The datasets used for analysis in the current study are available in NCBI datasets and can be accessed using the link provided: https://www.ncbi.nlm.nih.gov/datasets/genomes/?taxon=1773 (accessed on 21 May 2022).

## Supplementary Materials

The following are available online at https://www.mdpi.com/article/10.3390/molecules27196150/s1, Table S1: Details of the samples analyzed consisting of organism, accession IDs, annotations, and submission details. Table S2: A consolidated list of all the genes, their protein functions, and the corresponding pathways. The list consists of all types of mutations for each of the genes. Table S3: Details of 369 proteins annotated. Table S4: Details of amino acids mapped to individual surface patches of AAC2′. Table S5: Tabulation of compounds with their structure, name, and docking score.
