# Supplementary material for: Mycobacterium Time-Series Genome Analysis Identifies AAC2′ as a Potential Drug Target with Naloxone Showing Potential Bait Drug Synergism
Source: Molecules. 2022 Sep 20;27(19):6150. doi: 10.3390/molecules27196150 (PMC9571707; doi:10.3390/molecules27196150)
Supplement: Supplementary file 1 [file molecules-27-06150-s001.zip › Supp data/Table S5.pdf]

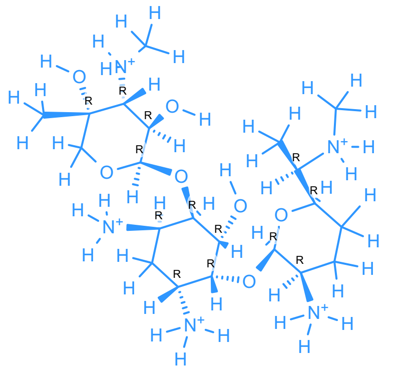

Source File	gentamicin.sdf
docking score	-6.036

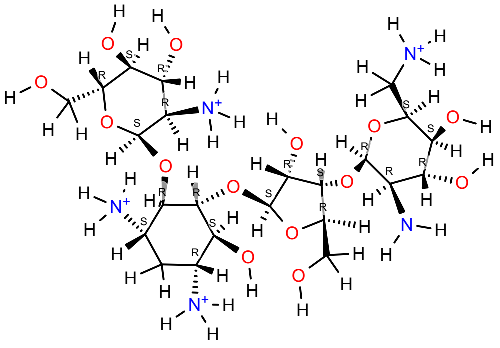

Source File	Paromomycin.sdf
docking score	-5.977

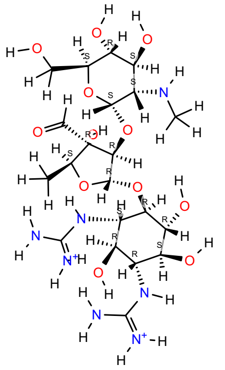

Source File	streptomycin.sdf
docking score	-5.907

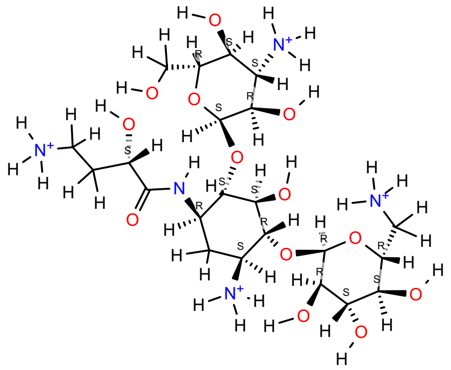

Source File	amikacin.sdf
docking score	-5.844

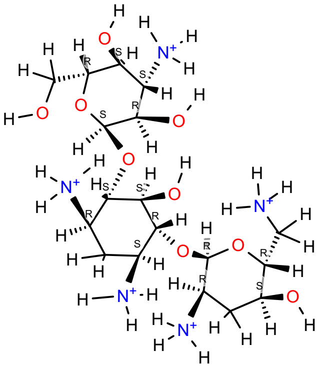

Source File	tobramycin.sdf
docking score	-5.733

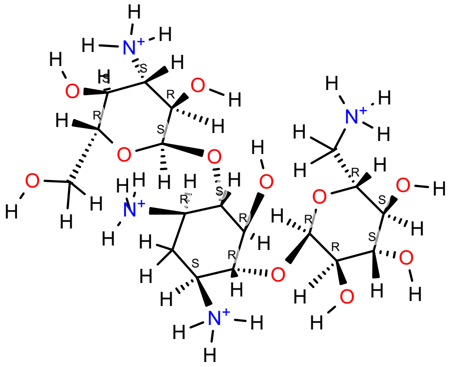

Source File	kanamycin.sdf
docking score	-5.65

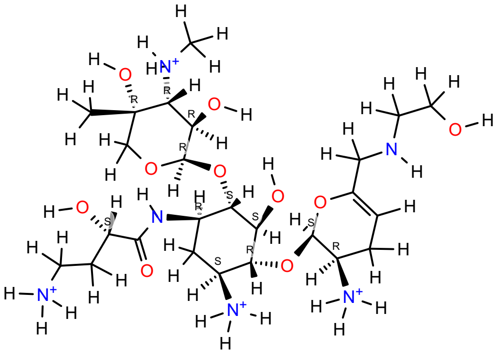

Source File	plazomicin.sdf
docking score	-5.328

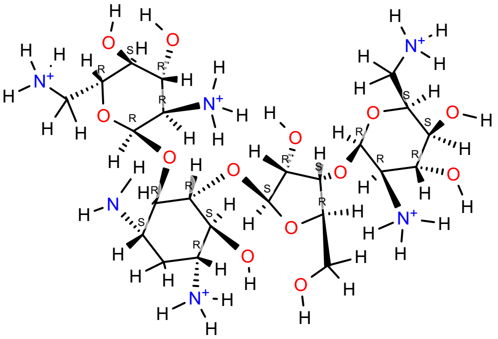

Source File	neomycin.sdf
docking score	-5.235

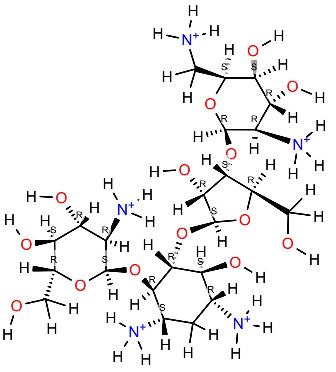

zinc_id	ZINC000060183170
docking score	-10.201

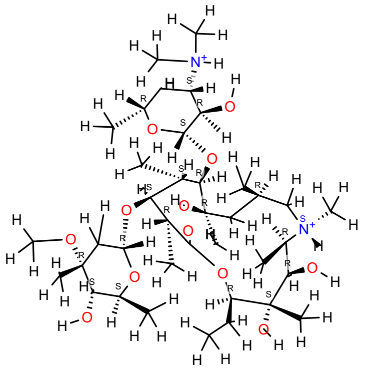

zinc_id	ZINC000085537026
docking score	-6.677

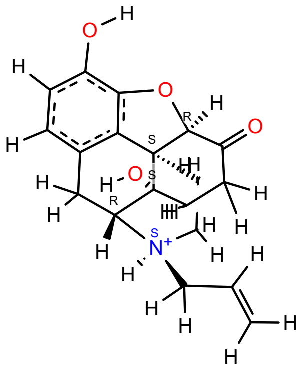

zinc_id	ZINC000000389747
docking score	-6.317

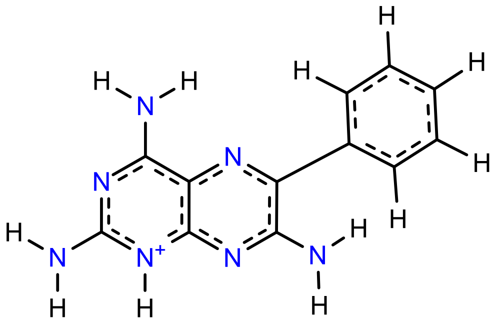

zinc_id	ZINC000000120286
docking score	-3.521
